# Should fluid dynamics be included in computer models of RF cardiac ablation by irrigated-tip electrodes?

**DOI:** 10.1186/s12938-018-0475-7

**Published:** 2018-04-20

**Authors:** Ana González-Suárez, Juan J. Pérez, Enrique Berjano

**Affiliations:** 1 Department of Information and Communication Technologies, Universitat Pompeu Fabra, Carrer Roc Boronat 138, 08018 Barcelona, Spain; 2 BioMIT, Department of Electronic Engineering, Universitat Politècnica de València, Valencia, Spain

**Keywords:** Blood flow, Cardiac ablation, Computer model, Irrigated electrode, Radiofrequency ablation, Thermal modeling

## Abstract

**Background:**

Although accurate modeling of the thermal performance of irrigated-tip electrodes in radiofrequency cardiac ablation requires the solution of a triple coupled problem involving simultaneous electrical conduction, heat transfer, and fluid dynamics, in certain cases it is difficult to combine the software with the expertise necessary to solve these coupled problems, so that reduced models have to be considered. We here focus on a reduced model which avoids the fluid dynamics problem by setting a constant temperature at the electrode tip. Our aim was to compare the reduced and full models in terms of predicting lesion dimensions and the temperatures reached in tissue and blood.

**Results:**

The results showed that the reduced model overestimates the lesion surface width by up to 5 mm (i.e. 70%) for any electrode insertion depth and blood flow rate. Likewise, it drastically overestimates the maximum blood temperature by more than 15 °C in all cases. However, the reduced model is able to predict lesion depth reasonably well (within 0.1 mm of the full model), and also the maximum tissue temperature (difference always less than 3 °C). These results were valid throughout the entire ablation time (60 s) and regardless of blood flow rate and electrode insertion depth (ranging from 0.5 to 1.5 mm).

**Conclusions:**

The findings suggest that the reduced model is not able to predict either the lesion surface width or the maximum temperature reached in the blood, and so would not be suitable for the study of issues related to blood temperature, such as the incidence of thrombus formation during ablation. However, it could be used to study issues related to maximum tissue temperature, such as the steam pop phenomenon.

## Background

Radiofrequency (RF) catheter ablation (RFCA) is a common and safe procedure used to eliminate cardiac arrhythmias in which RF current is delivered by a metal electrode embedded in the tip of a percutaneous catheter. The electrode design must be such that it achieves effective thermal necrosis of the target tissue (temperatures > 50 °C), while keeping its temperature below 80 °C to prevent the formation of thrombi in the blood [1]. Irrigated-tip electrodes have been proposed to meet both requirements [2]. These electrodes, which are in fact currently those most often used in clinical practice, allow continuous saline flushing through small holes distributed around the electrode tip to cool the blood-tissue interface [2].

Computer modeling is an analysis technique broadly used in studies on RFCA [3–12]. In order to obtain accurate results, the model has to be as realistic as possible, which implies ever more complicated mathematical formulations, especially in the case of modeling irrigated-tip electrodes for RFCA. In this case an accurate model should be based on a triple coupled problem involving simultaneous electrical conduction, heat transfer, and fluid dynamics [4], which is necessary to model the thermal effect of the circulating blood around the electrode placed on the endocardium and its interaction with the saline infused through the holes in its tip. Furthermore, the fluid dynamics problem forces the model to be three-dimensional, with the consequent additional computational cost. However, in certain cases it is difficult to have access to the adequate software and/or have the expertise necessary to be able to couple these three problems. Therefore, reduced models should be considered.

In this respect, a reduced model that dispenses with fluid dynamics is sometimes employed as an alternative [5–8]. In this case, the thermal cooling due to electrode irrigation is modeled by keeping a fixed temperature similar to that found in clinical practice in a zone of the electrode-tip. Although it has been suggested that this model could reproduce lesion depth reasonably well [8], the reduced and the full model have never been directly compared in terms of lesion dimensions and maximum temperatures reached in the blood and tissue. Our goal was thus to compare both models in terms of predicting thermal lesion dimensions and temperature distributions in blood and tissue. Since the full model had already been validated experimentally [4, 9], this study really sought to evaluate the worth of the reduced model by comparing its results with those obtained from the full model. In terms of clinical application, it is especially important that both models should accurately predict the lesion surface width and the maximum blood temperature achieved around the electrode tip, since it is known that these parameters are related to thrombus formation [1, 13, 14]. It is also important that they be able to predict the maximum temperature in the tissue, since values of around 100 °C are associated with the formation of steam pops [15, 16].

## Methods

### Description of the model geometry

**Fig. 1:**
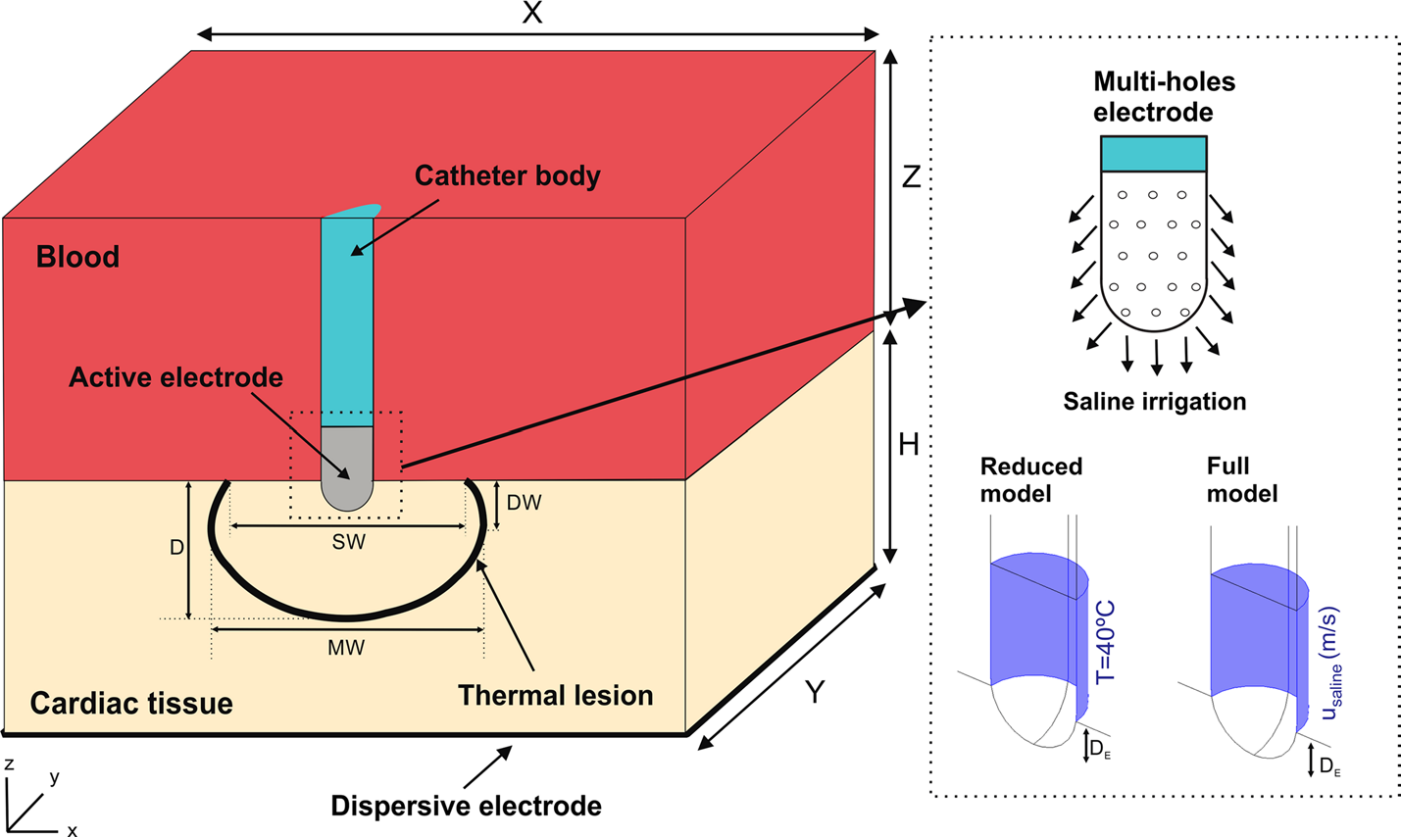
Geometry of the three-dimensional computational models built (not to scale). Cardiac tissue thickness (H) was 20 mm and the dimensions of cardiac chamber X and Y (Z = Y) were obtained from a convergence test. RF current flows between an active electrode and the dispersive electrode (bottom). The active electrode (7Fr, 4 mm) is a multi-hole open-irrigated electrode, which is assumed to be inserted into cardiac tissue to a depth D_E_. In the reduced model the saline irrigation through the small holes in the electrode tip is simply modeled by fixing a temperature condition of 40 °C on its surface, while in the full model it is modeled by an inlet velocity boundary condition U_saline_ at the electrode-blood interface. Thermal lesion is assessed by the 50 °C isotherm and its geometry is characterized by: maximum depth (D), maximum width (MW), depth at the maximum width (DW) and surface width (SW)

Figure 1 shows the geometry and dimensions of the three-dimensional computational model, where the XZ-plane is the symmetry plane. The model consists of a fragment of cardiac tissue and an open-irrigated electrode with dimensions (7Fr diameter and 4 mm length) similar to those used in clinical practice for RF cardiac ablation [17–20] surrounded by circulating blood (cardiac chamber). The electrode was placed perpendicular to the cardiac surface and inserted into the tissue to a depth D_E_, which varied in the simulations from 0.5 to 1.5 mm (no realistic details were required on the mechanical deformation of the tissue surface due to the electrode since the maximum insertion depth considered was assumed to be less than 2 mm [10]) similar to the range of penetration depths of RF ablation electrode at frequently ablated areas of the endocardium [21]. The open-irrigated electrode has multi-holes distributed around its entire distal tip, through which a saline solution is continuously flushing and mixing with the circulating blood, which were not individually included in the physical model as their extremely small size would have required an unnecessarily fine computational mesh. In the case of the reduced model, the saline irrigation was modeled by fixing a temperature at the electrode tip [5–8], while in the full model this was done using an inlet velocity boundary condition applied to the electrode surface where the irrigation holes are located [4]. The dispersive electrode was always modeled as an electrical boundary condition at a distance from the active electrode (bottom surface). Cardiac tissue thickness (H) was 20 mm [3, 4] and the remaining dimensions of the fragment of cardiac chamber (X, Y and Z) were estimated by means of a convergence test in order to avoid boundary effects. In this test, the value of the maximal temperature achieved in the tissue (T_max_) after 60 s of RF heating was used as control parameter. We first considered a tentative spatial (i.e. minimum meshing size) and temporal resolution. To determine the appropriate parameters of X and Y (Z = Y), we increased their values by equal amounts. When the difference in the T_max_ between consecutive simulations was less than 0.5%, we considered the former values to be adequate. We then determined adequate spatial and temporal resolution by means of similar convergence tests using the same control parameter as in the previous test. Discretization was spatially heterogeneous: the finest zone was always the electrode-tissue interface, where the largest voltage gradient was produced and hence the maximum value of current density. In the tissue, grid size was increased gradually with distance from the electrode-tissue interface.

### Governing equations

The numerical models were based on a coupled electric-thermal problem which was solved numerically using the finite element method (FEM) with COMSOL Multiphysics software (COMSOL, Burlington MA, USA). The Electric Current mode of the AC/DC module and the Heat Transfer module of COMSOL were used for the electric and the thermal problem, respectively. Simulations were run on a 64-bit PC with an eight-processor Intel Xeon platform running at 2.67 GHz with 48 GB of RAM. Simulations were run on a laptop Intel Core i7-4702MQ of 64-bits running at 2.20 GHz with 16 GB of RAM.The governing equation for the thermal problem was the Bioheat Equation modified by the enthalpy method [22], incorporating the phase change in order to model tissue vaporization:

1 $$\frac{\partial (\rho h)}{\partial t} = \nabla \cdot (k\nabla T) + q - Q_{p} + Q_{m}$$

where *ρ* was density (kg/m^3^), *h* enthalpy, *t* time (s), *k* thermal conductivity (W/m K), *T* temperature (°C), *q* the heat source caused by RF power (W/m^3^), *Q*_*p*_ the heat loss caused by blood perfusion (W/m^3^) and *Q*_*m*_ the metabolic heat generation (W/m^3^). *Q*_*p*_ was not considered since its effect is expected to be minor [7] and independent on the way used to model the saline irrigation and blood flow. Likewise, *Q*_*m*_ was not considered because its effect is negligible in comparison to the other terms [23]. In biological tissues, enthalpy is related to temperature by the following expression [22]:

2 $$\frac{\partial (\rho h)}{\partial t} = \frac{\partial T}{\partial t} \cdot \left\{ \begin{array}{l} \rho_{l} c_{l\,} \quad \quad \quad \quad 0 \le T \le 99\;^\circ {\text{C}} \hfill \\ H_{fg} C\quad \quad \quad \quad 99 < T \le 100\;^\circ {\text{C}} \hfill \\ \rho_{g} c_{g} \quad \quad \quad \quad T > 100\;^\circ {\text{C}} \hfill \\ \end{array} \right.$$

where *ρ*_*i*_ and *c*_*i*_ were the density and specific heat of cardiac tissue before phase-change (i.e. liquid phase, *i *= *l*) and post-phase-change (i.e. gas phase, *i *= *g*), respectively; *H*_*fg*_ was the latent heat and *C* the tissue water content. We considered a latent heat value of 2.162 × 10^9^ J/m^3^ which corresponds to the product of the water vaporization latent heat (2257 kJ/kg), the water density at 100 °C (958 kg/m^3^), and the tissue water content inside the cardiac tissue (75%).

At the frequencies used in RF heating (≈ 500 kHz) and over the distance of interest, the biological medium can be considered almost totally resistive, and a quasi-static approach can therefore be used to solve the electrical problem [24]. The distributed heat source *q* is then given by *q *=* σ|****E***|^*2*^, where |***E***| is the magnitude of the vector electric field (V/m) and *σ* the electrical conductivity (S m^−1^). ***E*** = − ∇Ф is calculated from the gradient of the voltage Φ (V), which, in absence of internal electric sources, satisfies ∇·(*σ*∇Ф) = 0.

### Model properties and boundary conditions

The thermal and electrical properties of the model elements are shown in Table 1 [3]. The initial model temperature was 37 °C, except in the electrode tip which was 22 °C in order to simulate the initial temperature of an open-irrigated electrode caused by the saline irrigation (which is kept at ambient temperature) [4]. The electrical (*σ*) and thermal conductivity (*k*) of cardiac tissue were temperature-dependent piecewise functions: for *σ* we considered an exponential growth of + 1.5%/°C up to 100 °C [11], where *σ*_*o*_ was the value of the electrical conductivity assessed at 37 °C (see Table 1), and then was reduced by 4 orders for five degrees to model the tissue desiccation process [25]; and *k* grew linearly 0.12%/°C up to 100 °C, *k*_*o*_ being the value of the thermal conductivity assessed at 37 °C (see Table 1), after which *k* was kept constant [26].

**Table 1:** Thermal and electrical characteristics of the elements employed in the models [3]: *σ*, electrical conductivity; *k*, thermal conductivity; *ρ*, density; and *c*, specific heat

Element/material	*σ* (S/m)	*k* (W/m K)	*ρ* (kg/m^3^)	*c* (J/kg K)
Electrode/Pt–Ir	4.6 × 10^6^	71	21,500	132
Catheter/Polyurethane	10^−5^	0.026	70	1045
Cardiac Chamber/Blood	0.667	0.541	1000	4180
Myocardium/Cardiac tissue				
Liquid phase	*σ*_*o*_^a^	*k*_*o*_^a^	1060	3111
Gas phase				
			370.44	2155.92

^a^*σ*_*o*_ and *k*_*o*_ were assessed at 37 °C: *σ*_*o*_ = 0.541 S/m and *k*_*o*_ = 0.531 W/m K

**Fig. 2:**
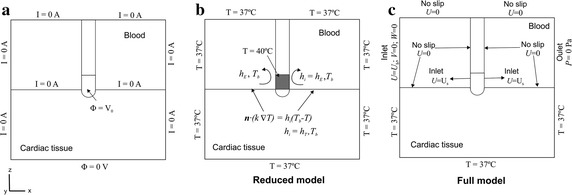
Boundary conditions of the models: **a** electrical boundary conditions, **b** thermal boundary conditions for the reduced model, and **c** thermal and velocity boundary conditions for the full model

Figure 2a shows the electrical boundary conditions. We modeled a constant power of 10 W for 60 s, which is the usual ablation mode for open-irrigated electrodes in RFCA. Note that the power level used in the numerical model was much lower than that used in clinical practice (around 35 W), since the entire volume of the patient’s body was not considered in the model, the impedance of the model thus being lower than in a real scenario [27]. We implemented a standard proportional-integral (PI) control algorithm using MATLAB (MathWorks, Natick, MA, USA) [28] and simulated it by means of the COMSOL-MATLAB interface, to which we had previously exported the FEM structure generated on COMSOL. The behavior of the PI controller was determined by the proportional (*K*_*p*_) and the integration (*K*_*i*_) constant, as described in detail by Haemmerich and Webster 2005 [28]. We adjusted the standard PI controller to keep the applied voltage to maintain the delivering power within 3% of the target, as electrical conductivity changes with increasing temperature (see Fig. 1 in the study conducted by Jain and Wolf 1999 [29]). We found that the parameters used in a previous study (*K*_*p*_ = 4.78, *K*_*i*_ = 3.39) were the best choice to reach the goal [4]. A Dirichlet voltage boundary condition was therefore applied on the ablation electrode surface. This voltage was calculated from the electrical current in each time-step obtained by the integration of the electrical density on the bottom surface (note that the model’s impedance was also estimated by this calculated electrical current). All the outer surfaces of the model (except the bottom surface) were fixed to a null electric flux (Neumann boundary condition). The voltage on the bottom surface was set to 0 V (dispersive electrode) to mimic a monopolar configuration in which RF current was forced to flow between the active and dispersive electrodes.

Figure 2b, c show the thermal and velocity boundary conditions. The effect of blood circulating inside the cardiac chamber and the saline irrigation were differently modeled for the reduced and the full model (see following subsection). In both cases, a null thermal flux was used on the symmetry plane and a constant temperature of 37 °C was fixed on the outer surfaces of the model.

### Modeling the blood motion and saline interaction

#### Reduced model

The effect of blood circulating inside the cardiac chamber was modeled by thermal convection coefficients at the electrode–blood (*h*_*E*_) and the tissue–blood (*h*_*T*_) interfaces (see Fig. 2b), considering electrical conductivity of blood independent of temperature (as in Method 2 described by [3]). Each coefficient was calculated under conditions of high and low blood velocity flow, the value of blood velocity being 8.5 and 3 cm/s for the high and low flow rates, respectively [12]. From these velocity values we obtained values of *h*_*E*_ = 3346 W/m^2^ K and *h*_*T*_ = 610 W/m^2^ K for high blood flow, and *h*_*E*_ = 2059 W/m^2^ K and *h*_*T*_ = 265 W/m^2^ K for low blood flow [3].

The effect of saline irrigation through the holes at the electrode tip was modeled by fixing a constant temperature of 40 °C only in the cylindrical zone of the electrode tip (see Fig. 2b), leaving the semispherical tip inserted into the tissue free, as in previous computational studies [5–8]. This temperature value was chosen due to its similarity with that obtained with multi-hole electrodes in clinical practice [30].

#### Full model

The effect of the interaction of blood motion and saline flow inside the cardiac chamber was modeled by including fluid dynamics equations (CFD module of COMSOL Multiphysics) coupled with the heat transfer equation. The advection term [12] was therefore included on the right of Eq. (1), which represents the heat loss due to blood flow:

3 $$\frac{{\partial \left( {\rho h} \right)}}{\partial t} = \nabla \cdot (k\nabla T) + q - Q_{p} + Q_{m} - \rho c\varvec{u} \cdot \nabla T$$

where ***u***(x, y, z) is the blood velocity field (m/s) and is derived by the incompressible Navier–Stokes equation, which consists of a Momentum equation (momentum principle or balance of forces) and a Mass equation (mass conservation):

4 $$\rho \frac{{\partial \varvec{u}}}{\partial t} + \rho \varvec{u} \cdot \nabla \varvec{u} = - \nabla P + \mu \nabla^{2} \varvec{u} + \varvec{F}$$

5 $$\nabla \cdot \varvec{u} = 0$$

where *P* is the pressure (Pa), *µ* the viscosity of the blood of 2.1 × 10^−3^ kg/(m s) and ***F*** (*F*_*x*_, *F*_*y*_, *F*_*z*_) the body forces (N/m^3^), which were neglected in our model.

Figure 2c shows the velocity boundary conditions applied to model the interaction of blood motion and saline flow. A no slip condition (non permeable surface, i.e. the fluid at the wall was not moving) was applied on the upper surfaces of the fluid volume and at the tissue-blood and electrode-blood interfaces. An inlet velocity boundary condition was applied to the left surface of the fluid volume to simulate the two blood velocities (in *x*-direction) of 8.5 and 3 cm/s for high and low flow rate, respectively. An outlet boundary condition of zero pressure was fixed on the right surface of the fluid volume. The saline irrigation flow was taken into account by an inlet velocity condition in the blood region, applied to a specific part of the electrode-blood interface surface where the holes were located, except in the part of the electrode tip inserted in the tissue [4]. The saline velocity condition was calculated as the ratio between the saline irrigation flow rate and the electrode area through which the saline flows (see violet zone in Fig. 1). We considered a saline irrigation flow rate of 8 mL/min, since this is the clinical value recommended by the manufacturer for a multi-hole electrode using power levels below 30 W [31]. The electrode area in which the saline velocity was applied changed according to the depth of electrode insertion into the tissue (D_E_): 0.0105, 0.0123 and 0.0147 m/s for values of D_E_ of 0.5, 1 and 1.5 mm, respectively.

### Output variables

As in previous studies, the thermal lesion shape was assessed by the 50 °C isotherm [3–7, 11], which is usually considered to reasonably represent the contour of irreversible myocardial injury in hyperthermic ablation. The thermal lesion shape was characterized using the following values (see Fig. 1) [2, 4, 31, 32]: maximum depth (D), maximum width (MW), depth at the maximum width (DW), and surface width (SW). We compared the thermal lesion dimensions and the maximum temperature values reached in the tissue and blood computed by the reduced model with those obtained by the full model. We also compared the lesion volumes at the end of ablation (60 s) computed using the formula described by [32]. We used the full model as a “ground truth” for comparison with the reduced model, since the full model had previously been validated against experimental data [4, 9]. We considered that the differences in lesion dimensions between both models were insignificant for values lower than 1 mm, since this value is approximately that of the deviation (± 0.5 mm) observed in experimental RFCA studies [3]. Likewise, the differences in maximum temperature reached in tissue and blood were considered to be insignificant for values lower than 4 °C, since this value is approximately that of the observed deviation (± 2 °C) [3].

## Results

After the convergence tests conducted with the computational model we obtained the following optimum values: dimensions X = 80 mm and Y = 40 mm (Z = Y), grid size of 0.2 mm in the finest zone (electrode-tissue interface), and step time of 0.05 s. The model had nearly 50,000 tetrahedral elements.

**Fig. 3:**
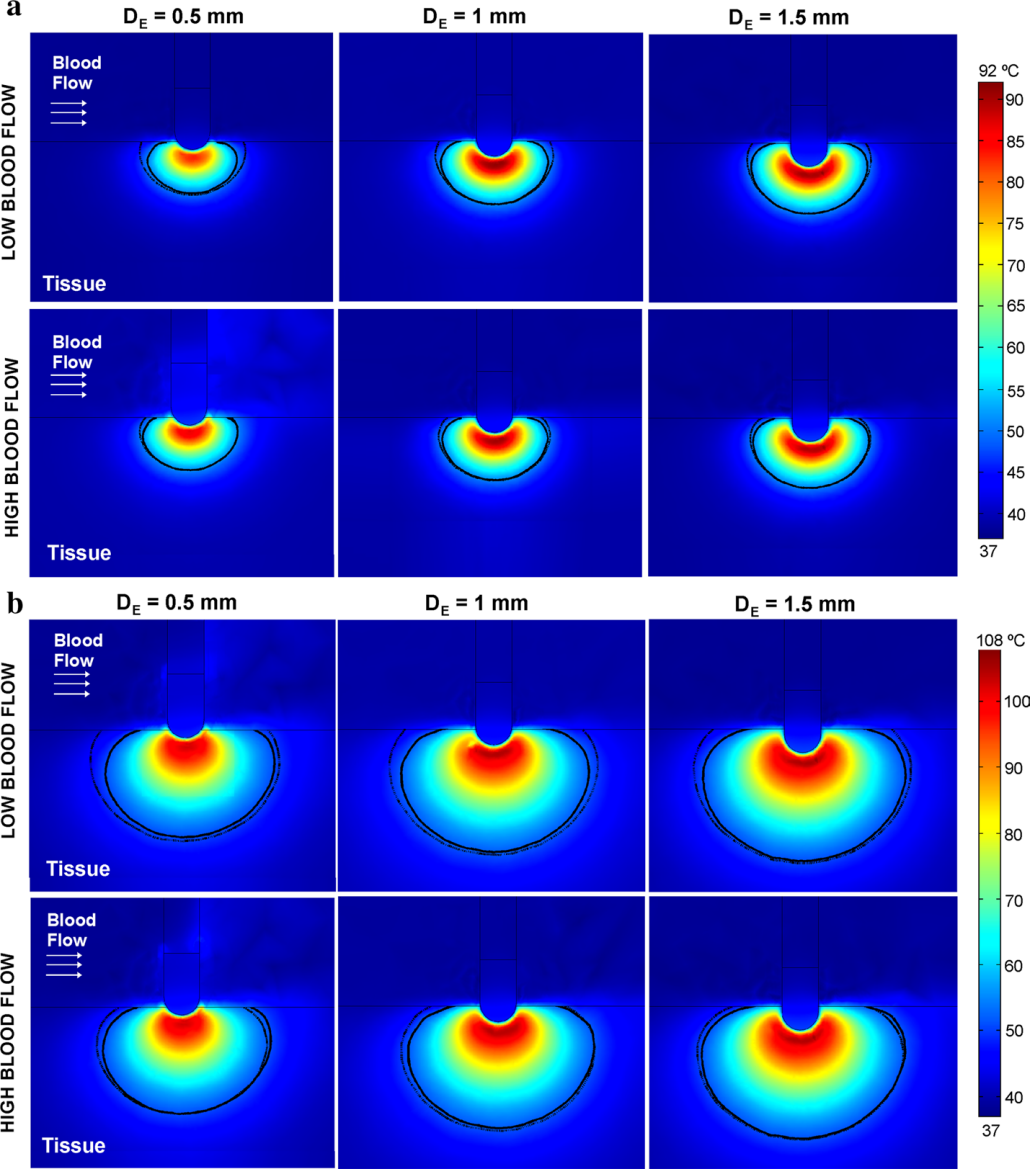
Temperature distributions at 10 s (**a**) and 60 s (**b**) obtained with the full model. Solid lines are those of the lesion boundaries computed by the full model while dashed lines are those computed by the reduced model

**Fig. 4:**
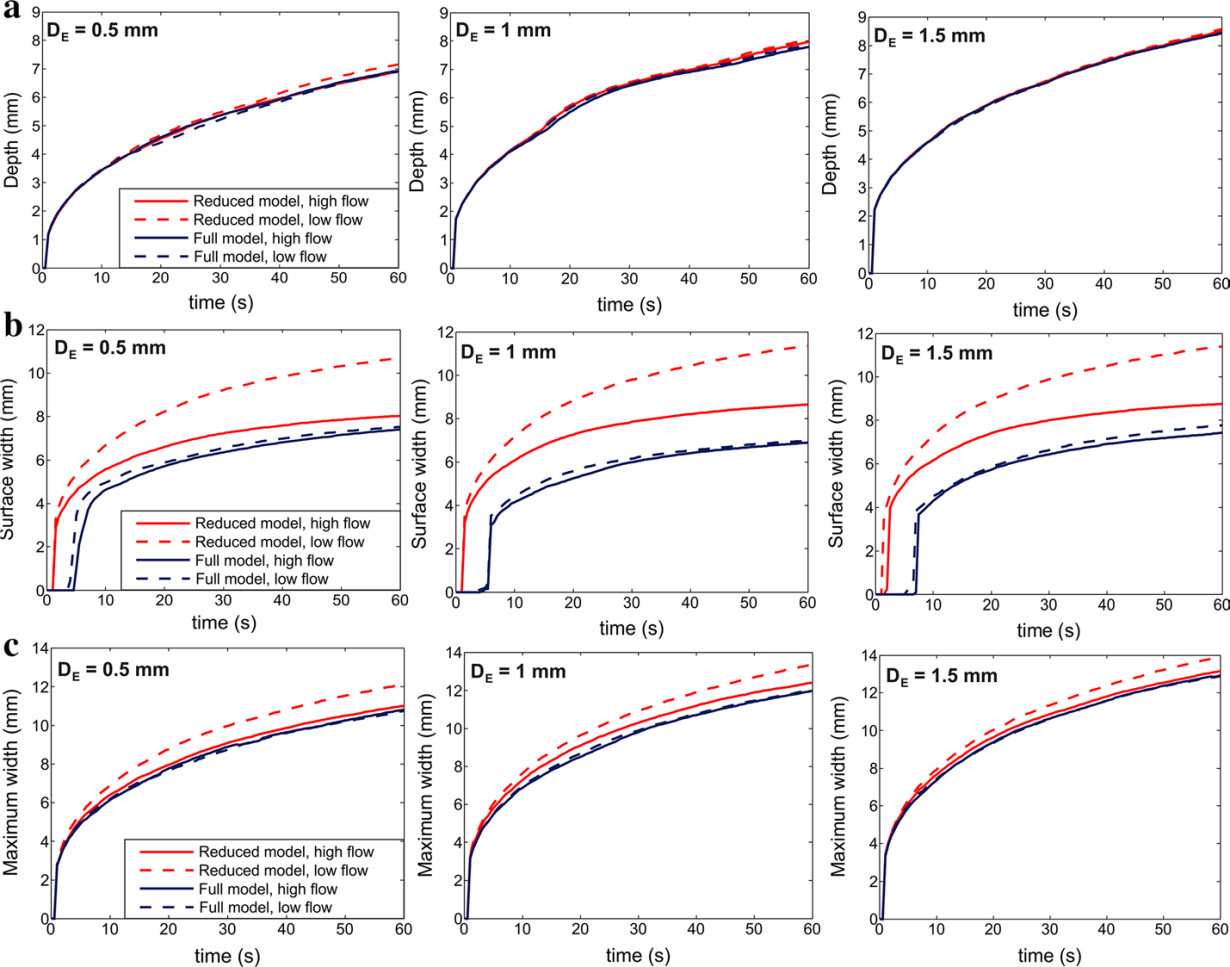
Comparison of the progress of lesion depth (**a**), surface width (**b**) and maximum width (**c**) between the reduced (red lines) and the full (blue lines) models, for high and low blood flow rates and three electrode insertion depths (D_E_) (0.5, 1.0 and 1.5 mm)

Figure 3 shows the temperature distribution obtained with the full model and the thermal lesion contours computed by both the full and the reduced model. It can be seen that the latter is not able to reproduce the typical ellipsoidal shape of the lesions created with irrigated-tip electrodes, which are characterized by a maximum width at some distance from the tissue surface and a minimum surface width. Indeed, as Fig. 4 shows, the reduced model fails to predict the lesion surface width, overestimating it by 5 mm in comparison with the full model (i.e. up to 70%) for any electrode insertion depth, and especially at low blood flow rates. Above all, it was noticeable that the surface lesion width is really created some seconds after starting the RF ablation (see progress in the full model), while the reduced model predicts surface width incorrectly, since the lesion is created almost from the beginning of RF ablation. Neither can the reduced model accurately predict the maximum width at low blood flow rates (differences around 1.5 mm with respect to the full model, i.e. ~ 15%). In fact, in this condition the reduced model fails to predict the lesion volume, as can be seen in Table 2. The lesion volume computed by the reduced model was always larger than that obtained by the full model, especially in the case of low blood flow, regardless of the electrode insertion depth. However, lesion depth is correctly predicted by the reduced model regardless of blood flow rate and electrode insertion depth (differences lower than 0.1 mm between both models, i.e. < 5%) and this predictive capacity is maintained throughout the entire ablation time (see Fig. 4a).

**Table 2:** Lesion volume (mm^3^) computed at 60 s for both models and different conditions of blood flow and insertion depths

Model	Blood flow	Insertion depth (mm)		
		0.5	1.0	1.5
Reduced	Low	390.2	516.2	593.1
	High	295.3	434.6	544.4
Full	Low	302.3	408.7	550.7
	High	290.5	400.7	526.8

**Fig. 5:**
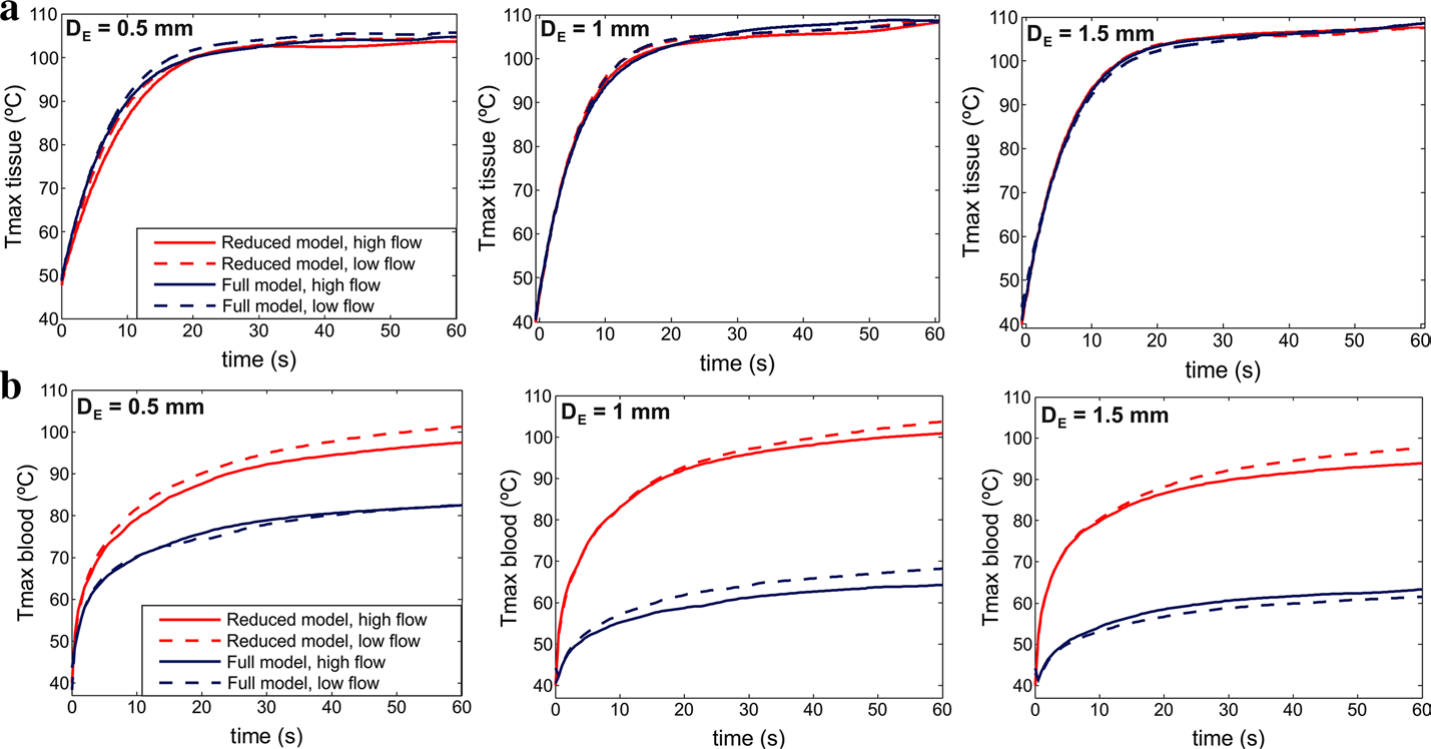
Comparison of the progress of maximum temperatures reached in the tissue (**a**) and blood (**b**) between the reduced (red lines) and full (blue lines) models, for high and low blood flow rates and three electrode insertion depths (D_E_) (0.5, 1.0 and 1.5 mm)

Figure 5 shows the progress of the maximum temperatures reached in the tissue and the blood for both models. The results show that the reduced model also fails to predict the maximum blood temperature reached in the proximity of the electrode-tissue interface, overestimating it drastically in all cases (differences between both models were always higher than 15 °C, and were even 35 °C higher at an electrode insertion depth of 1 mm). While the full model predicts that blood temperature will remain below 80 °C (which is the specific aim of irrigated-tip electrodes to prevent thrombus formation on the electrode surface), the reduced model wrongly predicts increases of up to 100 °C. This overestimation of maximum blood temperature makes the lesion surface width wider than that obtained by the full model, as shown in Fig. 3. On the other hand, the reduced model predicts the maximum temperature reached in the tissue reasonably well, regardless of blood flow rate and electrode insertion depth (differences always lower than 3 °C, i.e. ~ 3% for temperature around 100 °C). Moreover, the hot points in the tissue were located at approximately the same distance from tissue surface in both models (1, 1.5 and 2 mm for an electrode insertion depth of 0.5, 1 and 1.5 mm, respectively). Something similar happens with the lesion depth, as this capacity is maintained throughout the entire ablation time.

**Fig. 6:**
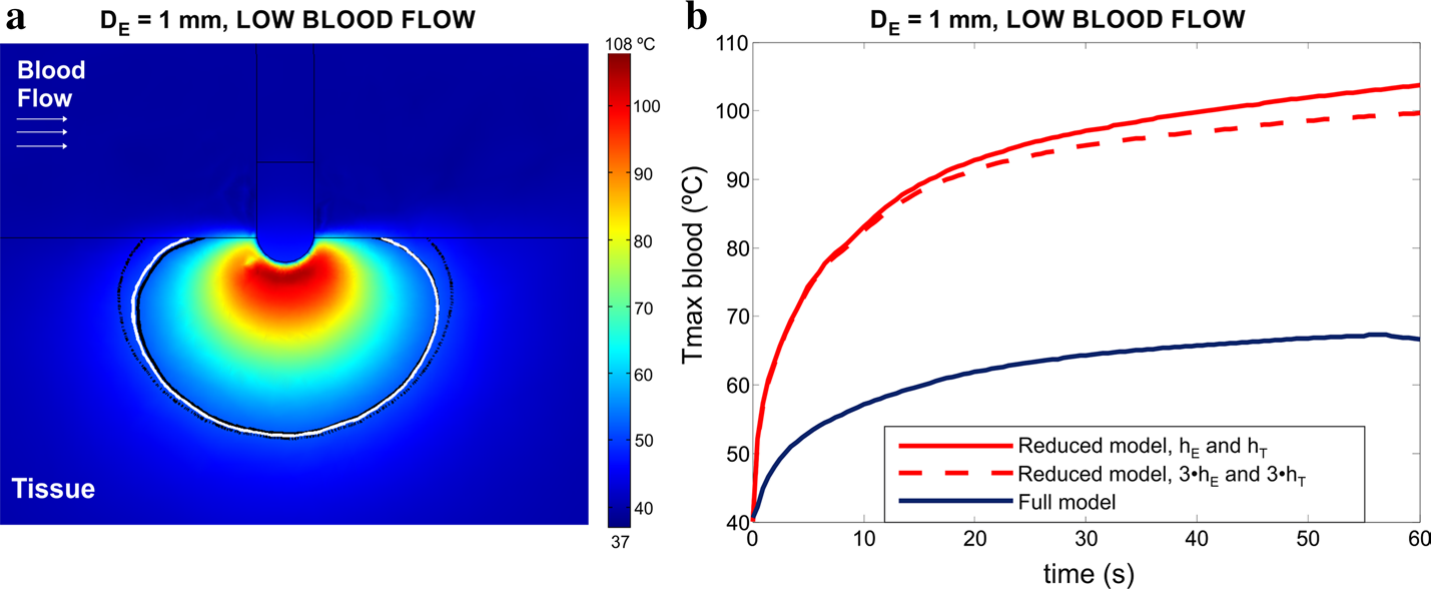
Temperature distributions at 60 s (**a**) for the full model (black and white solid lines are those of the lesion boundaries computed by the full and reduced model, in this case multiplying by three the thermal convective coefficients. The dashed line is that computed by the reduced model using the previous thermal convective coefficients) and the comparison of progress of maximum blood temperatures (**b**) between the reduced (red lines) and full (blue line) models for low blood flow rate using an electrode insertion depth (D_E_) of 1.0 mm. The rationale for increasing the value of thermal convective coefficients at both interfaces was to try to match the lesion surface width of the reduced model with that of the full model

In order to try to match the lesion surface width and the maximum blood temperature obtained with the reduced model with those achieved by the full model, we adjusted the thermal convective coefficients applied to the electrode-blood and tissue-blood interfaces (*h*_*E*_ and *h*_*T*_, respectively). We thus increased these coefficients to achieve a similar surface lesion width than that obtained with the full model. We considered one of the cases with the biggest difference in surface lesion width respect to the full model, for example the case of an electrode insertion depth of 1 mm with low blood flow. As can be seen in Fig. 6, a similar lesion surface width was obtained in both models by multiplying by three the thermal convective coefficients in the reduced model (see black and white solid lines), but the maximum blood temperature was still overestimated by more than 30 °C with respect to the full model.

## Discussion

This computer modeling study assessed the ability of a reduced model that does not consider fluid dynamics to compute the temperature distributions in tissue and blood during RFCA by irrigated electrodes in a comparison with the full model, which had previously been validated by experimental data [4, 9]. The reduced model would be promising if it could be used instead of the full model in certain circumstances, as it greatly simplifies certain issues such as geometry (often allowing a two-dimensional model instead of a three-dimensional model), mathematical formulation (reducing the amount of governing equations and boundary conditions, since only the electrical-thermal problem is solved without including fluid dynamics equations), considerably reducing the computational cost. Indeed, up to six times less computational time was required to solve the reduce model than for the full one (5.29 min vs. 31.73 min). Note that although the results from the reduce model were obtained with a three-dimensional model, they would have been the same with a two-dimensional model, further reducing the computational cost.

However, our results show that the reduced model is not suitable for studying temperature distributions in the blood; as can be seen in Fig. 5, the value of the maximum blood temperature in the proximity of the electrode-tissue interface was over-estimated, reaching a value of ~ 100 °C in all the cases, while the maximum blood temperature obtained with the full model always remained below 80 °C, as in a previous experimental work [33] (see Fig. 5 of that work for the cases using an ambient temperature for saline irrigation, as in our case). Our findings also showed that the reduced model cannot adequately predict either surface lesion width or lesion volume, since the former was wider than that achieved with the full model (see Fig. 3).

Although it was possible to adjust the surface lesion width obtained with the reduced model by increasing the thermal convection coefficients applied at the electrode-blood and tissue-blood interfaces to simulate the effect of circulating blood, it was impossible to obtain a realistic blood temperature distribution in the vicinity of the electrode-tissue interface (the maximum blood temperature still reached approximately 100 °C, very different from that of the full method, as can be seen in Fig. 6).

On the other hand, our results showed that the reduced model is able to accurately predict lesion depth at all times during ablation (up to 60 s), and also the maximum temperature reached in the tissue. In practical terms, a modeling study focusing on issues related with overheating occurring in the tissue (e.g. steam pops which are associated with intra-tissue temperatures of around 100 °C) could benefit from the results obtained with the reduced model, since they show the reliability of the full model as long as there is no blood circulating around the electrode-tip.

This study characterized the differences between both models under different electrode insertion depths and blood flow rates. Had other conditions (such as variations in tissue properties, the arrangement of the infusion holes on the electrode surface, and contact angle between electrode and tissue surface) also been included we think that the conclusions reached would still have been valid. In future work, computer models offering a realistic distribution of cardiac flow [34–39] could be coupled to the full model in order to increase their accuracy.

## Conclusions

The findings confirm that a reduced model that does not include the fluid dynamics is not suitable for predicting either temperature distributions in the blood or surface lesion width, which rules it out as a means of studying the factors involved in thrombi formation. The full model, which solves electrical conduction, heat transfer, and fluid dynamics simultaneously, should therefore be generally employed for simulating the performance of an RF irrigated electrode surrounded by circulating blood. The results indicate that both models give satisfactory results when predicting lesion depth and maximum tissue temperature, which indicates that the reduced model could be employed to study issues related to tissue temperature, such as the incidence of steam pops during ablation.
